# Diet-Induced Obesity Does Not Alter Tigecycline Treatment Efficacy in Murine Lyme Disease

**DOI:** 10.3389/fmicb.2017.00292

**Published:** 2017-02-24

**Authors:** Helena Pětrošová, Azad Eshghi, Zoha Anjum, Nataliya Zlotnikov, Caroline E. Cameron, Tara J. Moriarty

**Affiliations:** ^1^Matrix Dynamics Group, Faculty of Dentistry, University of TorontoToronto, ON, Canada; ^2^Department of Biochemistry and Microbiology, University of VictoriaVictoria, BC, Canada; ^3^Department of Laboratory Medicine and Pathobiology, Faculty of Medicine, University of TorontoToronto, ON, Canada

**Keywords:** *Borrelia burgdorferi*, Lyme disease, bacterial infection, antibiotics, tigecycline, obesity, diet-induced obesity, humoral response

## Abstract

Obese individuals more frequently suffer from infections, as a result of increased susceptibility to a number of bacterial pathogens. Furthermore, obesity can alter antibiotic treatment efficacy due to changes in drug pharmacokinetics which can result in under-dosing. However, studies on the treatment of bacterial infections in the context of obesity are scarce. To address this research gap, we assessed efficacy of antibiotic treatment in diet-induced obese mice infected with the Lyme disease pathogen, *Borrelia burgdorferi*. Diet-induced obese C3H/HeN mice and normal-weight controls were infected with *B. burgdorferi*, and treated during the acute phase of infection with two doses of tigecycline, adjusted to the weights of diet-induced obese and normal-weight mice. Antibiotic treatment efficacy was assessed 1 month after the treatment by cultivating bacteria from tissues, measuring severity of Lyme carditis, and quantifying bacterial DNA clearance in ten tissues. In addition, *B. burgdorferi*-specific IgG production was monitored throughout the experiment. Tigecycline treatment was ineffective in reducing *B. burgdorferi* DNA copies in brain. However, diet-induced obesity did not affect antibiotic-dependent bacterial DNA clearance in any tissues, regardless of the tigecycline dose used for treatment. Production of *B. burgdorferi*-specific IgGs was delayed and attenuated in mock-treated diet-induced obese mice compared to mock-treated normal-weight animals, but did not differ among experimental groups following antibiotic treatment. No carditis or cultivatable *B. burgdorferi* were detected in any antibiotic-treated group. In conclusion, obesity was associated with attenuated and delayed humoral immune responses to *B. burgdorferi*, but did not affect efficacy of antibiotic treatment.

## Introduction

Obesity is a complex metabolic condition that affects more than 13% of the world population (World Health Organization, 2015). The prevalence of obesity is even higher in North American countries, where almost 30% of the population is considered obese (Finucane et al., 2011). Obesity comorbidities include metabolic syndrome, cardiovascular disease and type 2 diabetes (Guh et al., 2009; O’Neill and O’Driscoll, 2015). Emerging evidence suggests that obesity is also associated with increased susceptibility to bacterial infections and severity of infection outcomes (Huttunen and Syrjänen, 2013). Obese patients are more frequently diagnosed with respiratory, skin and urinary infections, and are more prone to hospital-acquired and surgical-site infections (Waisbren et al., 2010; Kwong et al., 2011; Sreeramoju et al., 2011; Semins et al., 2012). Bacterial infections therefore significantly reduce quality of life among obese patients. However, studies on the treatment of bacterial infections in obese populations are scarce.

Successful treatment of infection relies on achieving the appropriate plasma and tissue drug concentrations over time that is required to inhibit or kill the pathogen. Antibiotic dosing is determined based on the antibiotic susceptibility of individual pathogens (minimal inhibitory and bactericidal concentrations; MIC and MBC) and the kinetics of antibiotic distribution and clearance from the body (pharmacokinetics). In obese patients, pharmacokinetic properties of antibacterial agents can be affected in numerous ways including decreased drug distribution due to partitioning of lipophilic drugs in enlarged adipose tissue, increased drug excretion caused by glomerular hyperfiltration, and increased drug volume distribution (Falagas and Karageorgopoulos, 2010). Antibiotic dosage can be adjusted based on the total body weight or ideal body weight, with hydrophilic antibiotics likely to be adjusted for the former and lipophilic for the latter (Falagas and Karageorgopoulos, 2010). Other factors that have to be considered when designing optimal dosing include antibiotic toxicity and induction of antibiotic resistance in bacteria when using subtherapeutic doses (pharmacodynamics) (Pai, 2015; Drusano et al., 2016). Therefore, antibiotic dosing in obesity is a complex problem.

Animal models of obesity recapitulate associated complications of obesity in humans, including features of metabolic syndrome (hypertension, hyperglycemia, hyperlipidemia, and hypercholesterolemia). Since human obesity often stems from excessive caloric intake and consumption of energy-dense meals, the animal model which best approximates human obesity is high fat diet-induced obesity (DIO) (Lutz and Woods, 2012). DIO mice exhibit an attenuated ability to control infection by *Staphylococcus aureus*, *Porphyromonas gingivalis* and the Lyme disease pathogen *Borrelia burgdorferi* (Amar et al., 2007; Yano et al., 2012; Farnsworth et al., 2015; Zlotnikov et al., 2016). We and others have recently reported that DIO, as well as severe obesity-independent hyperglycemia and obesity-independent hypercholesterolemia, in mice inhibit control of *B. burgdorferi* burden and/or tissue clearance of bacterial DNA, and are associated with altered innate and adaptive immune responses to this bacterium (Toledo et al., 2015; Javid et al., 2016; Zlotnikov et al., 2016). Thus, obesity and metabolic conditions associated with obesity can alter the outcomes of infection with the Lyme disease pathogen in mouse models.

Lyme disease is a tick-born infection that is increasingly common in many North American and European countries where obesity is highly prevalent (Finucane et al., 2011; Mead, 2015). Clinical manifestations of Lyme disease include a characteristic rash at the site of a tick bite (erythema migrans), inflammation of the heart and joints (Lyme carditis and Lyme arthritis, respectively) and neurological complications (neuroborreliosis). The most widely used animal model of Lyme disease, C3H mice, recapitulates manifestations of carditis and arthritis. However, similar to all other mouse models, C3H mice are resistant to skin and neurological manifestations. Lyme disease in humans is routinely treated with the antibiotics doxycycline and ceftriaxone, and there are no clinically relevant strains of antibiotic-resistant *B. burgdorferi* (Wormser et al., 2006). Currently, there are no specific guidelines for antibiotic treatment of Lyme disease in obese patients (Wormser et al., 2006; Borchers et al., 2015).

*Borrelia burgdorferi* DNA can be detected in tissues of infected hosts including humans, mice, dogs and non-human primates long after clinical symptoms of infection have resolved, as well as after antibiotic treatment (Nocton et al., 1994; Straubinger et al., 1997; Bockenstedt et al., 2002; Hodzic et al., 2008, 2014; Barthold et al., 2010; Yrjänäinen et al., 2010; Embers et al., 2012). There is controversy over whether this DNA derives from live bacteria or uncleared bacterial debris, and whether residual bacteria or their debris can continue to induce pathology in hosts (Wormser and Schwartz, 2009; Bockenstedt and Radolf, 2014). Interpretation of studies of this problem conducted in mouse models of Lyme disease are complicated by differences in the pharmacokinetic properties of the antibiotics used for clinical treatment of this disease, which differ in mice and humans (Wormser and Schwartz, 2009).

The tetracycline derivative tigecycline has a longer half-life in mice than doxycycline and ceftriaxone, and has been used to study *B. burgdorferi* persistence after infection and antibiotic treatment (Barthold et al., 2010). Tigecycline belongs to the class of lipophilic antibiotics, which generally require higher dosage to achieve appropriate serum concentrations in obese individuals (Falagas and Karageorgopoulos, 2010). Pharmacokinetic studies have found no effect of obesity on tigecycline serum concentration or urine clearance in healthy adult humans (Pai, 2014), but increased weight and body surface area are associated with increased tigecycline clearance in individuals with skin infections and pneumonia, many of whom have significant comorbidities (Wart et al., 2006; Rubino et al., 2010). The effects of obesity on tigecycline pharmacokinetics and efficacy in mice have not been studied.

We found recently that DIO in C3H/HeN mice is associated with attenuated innate immune responses to *B. burgdorferi*, reduced tissue clearance of *B. burgdorferi* DNA and increased severity of Lyme carditis (Zlotnikov et al., 2016). At a late acute stage of infection (4 weeks post-inoculation), the effect of DIO is most pronounced in female mice, where bacterial DNA burden in DIO was increased by ∼4-fold across all tissues, with ∼2–17-fold increases in burden observed in the brain, heart, knee joint, liver and lung (Zlotnikov et al., 2016). In the present study, we investigated whether efficacy of antibiotic treatment of *B. burgdorferi* infection is altered in the context of DIO in female C3H/HeN mice.

## Materials and Methods

### Ethics Statement

This study was carried out in accordance with the recommendations of the Guide to the Care and Use of Experimental Animals by the Canadian Council on Animal Care. The protocol was approved by the University of Toronto Animal Care Committee (permit #20011501). *Borrelia burgdorferi* was handled in accordance with guidelines of the University of Toronto, Public Health Agency of Canada and Canadian Food Inspection Agency (permit #12a-M30-2).

### Animals

**FIGURE 1:**
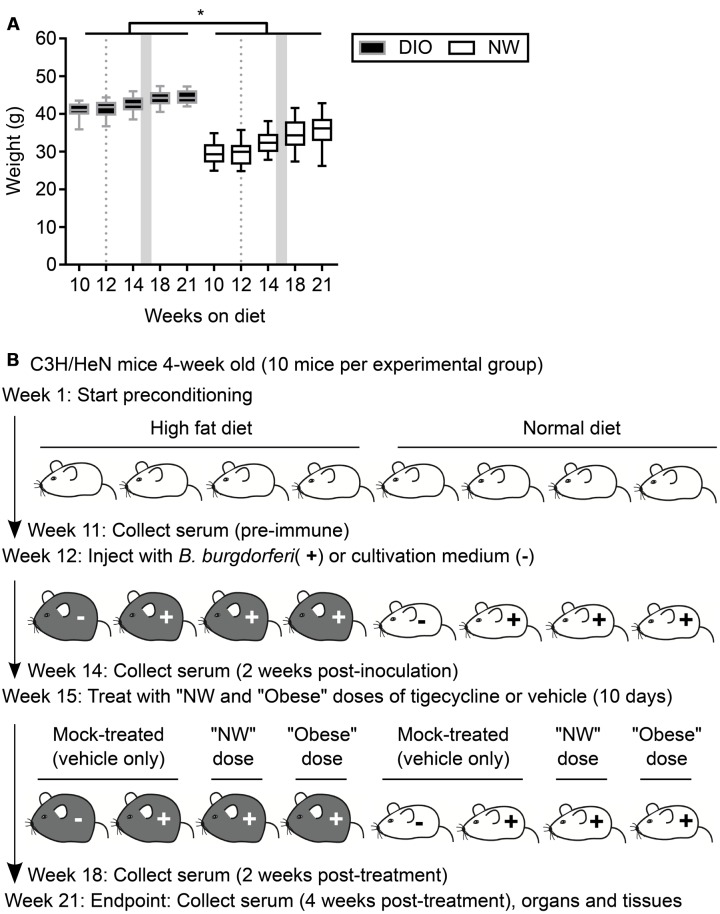
**Mouse model of antibiotic treatment of *B. burgdorferi* infection. (A)** Weights of mice were measured throughout the course of the experiment. DIO mice are shown in black (gray margins) and NW mice in white. Box and whiskers represent means with 95% confidence intervals (CI). Gray dotted lines and shaded areas represent the time of infection and the period of antibiotic treatment, respectively. ^∗^indicates *p* < 0.05 within individual time points (two-way ANOVA with Holm-Sidak post-tests). **(B)** Experimental scheme. DIO mice are depicted in gray, NW mice in white. “NW” and “obese” doses consisted of 12.5 mg/kg of tigecycline adjusted to the average weight of lean or DIO mice at the beginning of the treatment (32.5 and 42.7 g, respectively. See Material and Methods for details).

Four-week old female C3H/HeN mice were purchased from Charles River (Montréal, QC, Canada). Mice were housed in groups of four per cage under pathogen-free conditions with environment enrichment. Immediately upon arrival, mice were randomly assigned to one of two groups and fed *ad libitum* with high-fat diet (Teklad TD.06414, Harlan Laboratories, Inc., Mississauga, ON, Canada) or standard rodent chow (Teklad 2018 Rodent Chow, Harlan Laboratories). Mice preconditioned on high-fat diet received 18.4, 21.3, and 60.3% kcal from protein, carbohydrate and fat, respectively. Mice preconditioned on normal diet consumed 20.1, 69.8, and 10.2% kcal from protein, carbohydrate and fat, respectively. High-fat diet-fed mice were obese (DIO: body weight > 25% of weight of age-matched mice fed normal diet: normal weight, NW) (**Figure 1A**). All experimental animals, including mock-infected controls, were transferred to a biosafety level 2 (BSL2) room within the same animal care facility the day before infections and mock-infections, and were housed in this room for the remaining duration of experiments. Food, housing and environmental enrichments conditions were similar in BSL1 and BSL2 rooms. At the experimental end point, mice were anesthetized with 10 mg/kg xylazine (MTC Pharmaceuticals, Cambridge, ON, Canada) and 200 mg/kg ketamine hydrochloride (Rogar/STB, Montréal, QC, Canada), and sacrificed by cervical dislocation.

### *B. burgdorferi* Strains

For all following experiments, infectious B31 5A4-derived GFP-expressing *B. burgdorferi* strain was used (Moriarty et al., 2008). *B. burgdorferi* was grown in Barbour-Stoenner-Kelly (BSK-II) medium at 36°C and 1.5% CO_2_ (Barbour, 1984; Moriarty et al., 2012). Where needed, BSK-II was supplemented with 100 μg/ml gentamycin (Bioshop, Burlington, ON, Canada) (BSK-II-G) and 1X *Borrelia* antibiotic mixture containing 20 μg/ml phosphomycin (Sigma–Aldrich, Oakville, ON, Canada), 50 μg/ml rifampicin and 2.5 μg/ml amphotericin B (both from Bioshop). For all experiments, freshly inoculated *B. burgdorferi* cultures were grown to a logarithmic phase (less than 8 × 10^7^ cells/ml).

*Borrelia burgdorferi* gradually loses plasmids required for infectivity during *in vitro* passaging (Thomas et al., 2001). To ensure that *B. burgdorferi* used for experiments were fully infectious, we passaged the infectious B31 5A4-derived GFP-expressing *B. burgdorferi* strain GCB726 (Moriarty et al., 2008) through a mouse. A total number of 10^4^ bacteria in 100 μl BSK-II-G were subcutaneously injected at the dorsal lumbar midline of a C3H/HeN mouse. The mouse was sacrificed 3 weeks post-infection and *B. burgdorferi* were recovered from tibiotarsal joints by incubation in 5 mL BSK-II-G supplemented with 1X *Borrelia* antibiotic mixture. The resulting *B. burgdorferi* strain was denoted TMB79, passaged once, and frozen in glycerol at -80°C. TMB79 used for subsequent experimental infections was grown fresh from these glycerol stocks.

### Determination of Minimal Inhibitory and Bactericidal Concentrations of Tigecycline

To determine the concentrations of tigecycline to be used for treating *B. burgdorferi*-infected mice, we first determined the minimal inhibitory and bactericidal concentrations (MIC and MBC, respectively) for *B. burgdorferi* strain TMB79. To determine the MIC, twofold serial dilutions of tigecycline in BSK-II-G (from 50 to 0.1 mg/l) were prepared in a 96-well plate (Nunc/ThermoFisher Scientific, Mississauga, ON, Canada). Biological triplicates of *B. burgdorferi* logarithmic cultures were inoculated into the wells with antibiotics to a final concentration of 2 × 10^6^ cells/ml per well. Plates were incubated for 48 h and the number of motile *B. burgdorferi* per well was counted using a Petroff-Hausser chamber (Hausser Scientific, Horsham, PA, USA) and darkfield microscope. Non-motile bacteria were thinner, no longer luminous under darkfield microscopy and had disruptions in their normal sine wave morphology. The MIC was defined as the lowest tigecycline concentration at which the number of motile *B. burgdorferi* did not exceed the number of bacteria used for the initial inoculation, as previously described Barthold et al. (2010). To determine the MBC, 50 μl from each of the triplicate wells in MIC experiments were inoculated into 5 ml of BSK-II-G (total inoculum was 150 μl). The MBC was the lowest tigecycline concentration at which cultures remained negative (no *B. burgdorferi* present) after 21 days of incubation, as previously defined Barthold et al. (2010). All plates were kept at 36°C and 1.5% CO_2_ without shaking.

### Experimental Infections and Antibiotic Treatment

After 12 weeks of diet preconditioning, mice were randomly assigned to eight experimental groups with 10 mice per group. Three groups of DIO and three groups of NW mice were subcutaneously injected at the dorsal lumbar midline with 10^4^ *B. burgdorferi* (strain TMB79) in 100 μl of BSK-II-G. One group of DIO and one group of NW mice were injected with 100 μl of BSK-II-G medium alone (“mock-infected”). Three weeks post-inoculation, two groups of *B. burgdorferi*-infected DIO and two groups of *B. burgdorferi*-infected NW mice were treated daily for 10 consecutive days by intraperitoneal injection with 12.5 mg/kg tigecycline (LKT Laboratories Inc., St. Paul, MN, USA) (Barthold et al., 2010). One of each of the infected DIO and NW groups was treated with a “normal dose” of tigecycline, which was calculated based on the average weight of the NW group at treatment onset (32.46 g: **Figure 1A**). One of each of the infected DIO and NW groups was treated with “obese dose” tigecycline, which was calculated based on the average weight of the DIO group at treatment onset (42.65 g: **Figure 1A**). One of each of the infected DIO and NW groups was also treated over the same treatment period with vehicle only (“mock-treated”: 10% DMSO in 0.9% saline). Since tigecycline is highly unstable in saline (Barthold et al., 2010), antibiotic solutions were prepared daily from 10X stock solutions (filter-sterilized tigecycline in DMSO, frozen at -20°C in single-use aliquots). The experimental scheme is visually summarized in **Figure 1B**.

### Measurement of *B. burgdorferi* DNA Copy Number in Target Tissues

DNA was isolated from heart, patella (knee joint), quadriceps muscle, bladder, ear, skin, brain, liver, and lung from sacrificed animals using PureLink Genomic DNA Mini Kit according to manufacturer’s instructions (ThermoFisher Scientific). Isolated DNA was used as a template for quantitative real-time PCR (qPCR) measurement of *B. burgdorferi flaB* DNA copy number, as recently described (Javid et al., 2016). Runs with *R*^2^ values lower than 0.9 and/or amplification efficiency that was not within the range of 85 – 120 % were repeated. Each genomic DNA sample was run in six technical replicates. *flaB* DNA copy numbers in each sample were quantified using a standard curve consisting of technical duplicates of 10-fold serial dilutions of plasmid DNA pTM222 (Lee et al., 2010) containing a known copy number of *flaB* DNA (10^7^ – 1 copies). After inspection of all melt curves to identify and eliminate all products without the correct melting temperature for the *flaB* amplicon, the average copy number of *flaB* DNA in each sample was normalized to the DNA concentration of the same sample to control for possible differences in DNA extraction efficiency. To control for possible false positive results (stemming from stochastic amplification and/or amplification of non-*B. burgdorferi flaB* DNA sequences with a similar amplicon melting profile from tissues), we set a threshold for each tissue by calculating the mean amplification value for the corresponding tissue isolated from mock-infected animals plus two times standard deviation of the mean for mock-infected samples. Samples were considered qPCR-positive if the average *flaB* copy number was higher than the threshold.

### Histology and Carditis Scoring

Sagittal sections of hearts harvested from sacrificed mice were placed in 10% neutral buffered formalin (Sigma Aldrich), which was changed after 24 h of fixation. Samples were subsequently embedded in paraffin, sectioned and stained with hematoxylin and eosin (H&E; Toronto Centre for Phenogenomics, Toronto, ON, Canada). Carditis scoring was performed using a modified protocol for quantifying multifocal cardiac inflammation (Diebold et al., 1995), as recently described Javid et al. (2016) and Zlotnikov et al. (2016). The numbers of nuclei in five 100 mm^2^ regions of interest (located in each atrium and ventricle and the heart apex) in 3 matched sagittal sections were enumerated using a counting grid and averaged for each heart. The majority of each region of interest consisted of myocardium.

### Serum Collection and Measurement of Anti-*B. burgdorferi* IgM and IgG Titers

Approximately 100 μl of blood was collected from saphenous veins of mice using microvettes (Sarstedt, Montréal, QC, Canada) at three different time points: pre-immune (1 week before *B. burgdorferi* inoculation), post-inoculation (2 weeks after *B. burgdorferi* inoculation) and post-treatment (5 weeks after *B. burgdorferi* inoculation, 2 weeks after last antibiotic treatment). Serum was collected after spinning microvettes for 5 min at 10,000 × g at room temperature (22–25) (Microfuge 22R centrifuge with fixed-angle rotor, Beckman Coulter, Mississauga, ON, Canada), and stored immediately at -80°C until thawing for analysis. Serum was also collected from blood obtained by cardiac punctures at the experimental endpoint (7 weeks after *B. burgdorferi* inoculation, 4 weeks after last antibiotic treatment), and processed in the same manner.

*Borrelia burgdorferi* cells were harvested, resuspended to a final density of 1 × 10^9^ cells/ml, and processed as described previously (Lourdault et al., 2009; Eshghi et al., 2015). Ninety-six-well ELISA plates (Nunc-Immuno/ThermoFisher Scientific) were coated with 100 μl of the *B. burgdorferi* whole cell lysate. All incubation steps were performed at 37°C for 1 h, and were followed by washing with 0.05% Tween 20 in 1X phosphate-buffered saline (PBS-T). Firstly, plates were incubated with blocking buffer (0.5% skim milk in PBS-T). Secondly, diluted mouse serum (1:100 or 1:700 dilutions) was added to each well in technical duplicates. Finally, plates were incubated with 1:10,000 diluted HRP-tagged goat anti-mouse IgG antibody (Abcam, Toronto, ON, Canada). To detect bound secondary antibodies, ABTS substrate (Roche, Mississauga, ON, Canada) was added to each well. After 1 h incubation at room temperature, absorbance was measured at 405 nm (A_405_; for detection of the ABTS conversion) and 492 nm (A_492_; reference wavelength for detection of non-specific products) using a CLARIOStar plate reader (BMG LabTech, Guelph, ON, Canada). Final IgG concentrations were plotted as A_405_ values corrected by subtraction of blank values (A_405_ values of wells not coated with *B. burgdorferi* whole cell lysate and processed as described above), and reference A_492_ values. Concentrations of *B. burgdorferi* specific IgMs were determined following the protocol described above using pooled individual serum samples from each experimental group (diluted 1:100) and HRP-tagged goat anti-mouse IgM antibodies (Abcam) at 1:10,000 dilution. Normal mouse serum and serum from untreated *B. burgdorferi*-infected mice were used on every plate as negative and positive controls, respectively.

### Statistical Analysis

**FIGURE 2:**
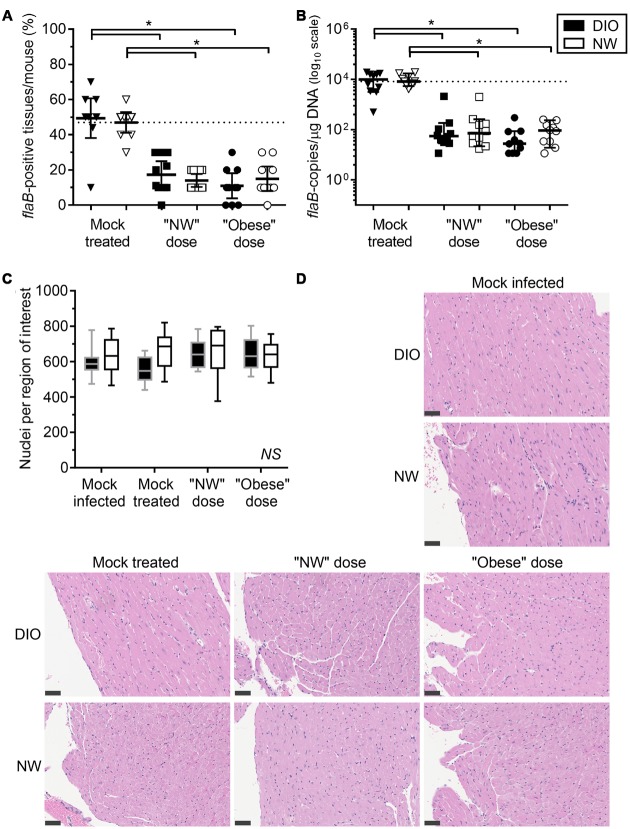
**Diet-induced obesity has no effect on tissue clearance of *B. burgdorferi* in antibiotic-treated mice**. Bacterial DNA in tissues of mice was quantified by qPCR with primers specific to the *B. burgdorferi flaB* gene. DIO mice are shown in black and NW mice in white. Individual values from mock-treated groups are depicted as triangles. Squares and circles stand for values measured in groups treated with “NW” and “Obese” doses of tigecycline, respectively. **(A)** Percentage of qPCR-positive tissues per mouse. Dotted line represents the average percentage of positive tissues in NW mock-treated mice (47%). Bars correspond to means with 95% CI. **(B)** *flaB* DNA copy number in all tissues of individual mice, calculated from individual copy numbers measured in ear, bladder, skin, knee joint (patella), brain, kidney, quadriceps, heart, lung and liver. Copy number is normalized to 1 μg of total host and bacterial DNA in each sample (data for individual tissues are provided in Supplementary Figures S1 and S2). Dotted line represents the median *flaB* DNA copy number in tissues of mock-treated NW mice (8.4ˆ10^3^ *flaB* DNA copies/μg DNA). Bars display medians with 95% CI. **(C,D)** Carditis severity. Sagittally hemisected hearts were fixed and stained by hematoxylin and eosin (H&E). **(C)** Numbers of nuclei per 100 mm^2^ region of interest, calculated from counts obtained in five regions of interest (each atrium and ventricle and the heart apex) in three matched H&E stained sections of heart. DIO mice are shown in black (gray margins) and NW mice in white. Means with 95% CI are shown. **(D)** Representative heart sections. Bars correspond to 50 μm (40x magnification). ^∗^indicates *p* < 0.05 (two-way ANOVA with Holm-Sidak post-tests; values in **(B)** were log-transformed prior to statistical analysis). *NS*, no significant differences among any groups (two-way ANOVA with Holm-Sidak post-tests).

**FIGURE 3:**
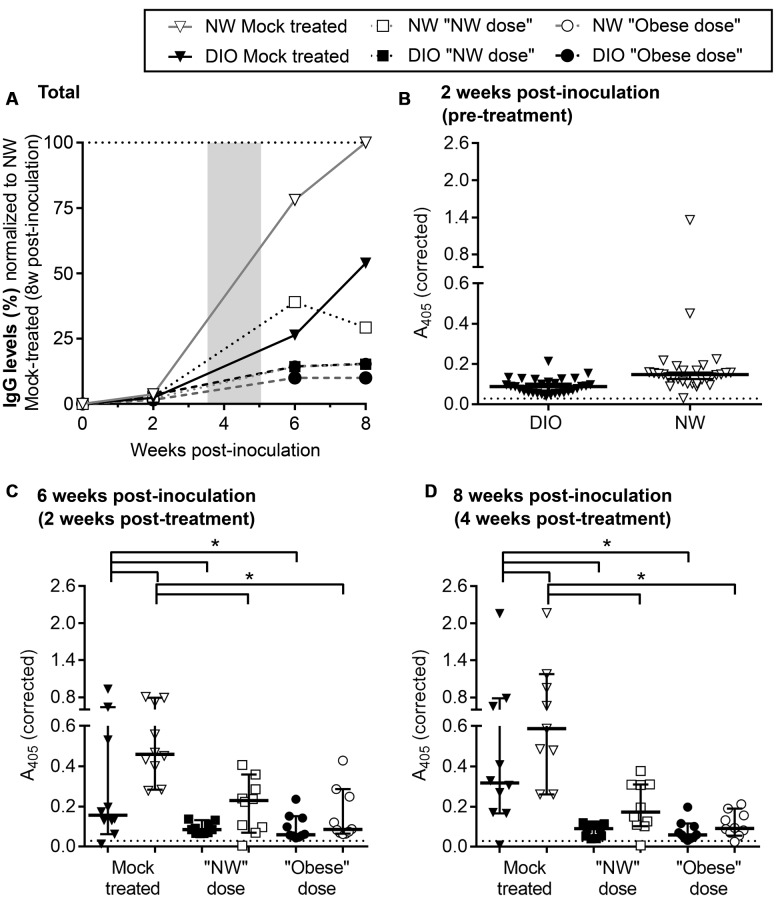
**Reduced *B. burgdorferi*-specific serum IgGs in DIO mice**. **(A)** Development of *B. burgdorferi*-specific IgG responses in DIO and NW mice during infection and tigecycline treatment. DIO and NW mice are depicted as black and white shapes (with black and gray lines, respectively). Mice from mock-treated groups are represented by triangles. Mice treated with “NW” and “Obese” doses of tigecycline are depicted as squares and circles, respectively. The shaded area represents the period of antibiotic treatment. To facilitate visual comparison among groups and time points, serum dilution-corrected IgG levels were normalized to median IgG levels measured in NW mock-treated mice 8 weeks post-infection (100%; dotted line). Shown are median values in each group. Raw values which were not corrected for serum dilution or normalized to the NW mock 8 week control group are shown in **(B–D)**. Non-normalized A_405_-corrected absorbance values for IgG ELISAs conducted with sera collected pre-treatment (**B**: 2 weeks post-inoculation), and at 2 and 4 weeks post-treatment (**C**,**D**: 6 and 8 weeks post-inoculation). Serum samples were diluted 1:100 **(B)** or 1:700 **(C,D)** and tested against whole cell lysate of *B. burgdorferi* strain that was used for infection. Shown are individual values, medians with 95% CI. ^∗^indicates *p* < 0.05 (two-way ANOVA with Holm-Sidak post-tests). Pre-immune sera from individual mice were used as a negative control at 1:100 dilution (dotted lines in **B–D**). There was no difference between pre-immune (non-specific) IgG levels of DIO and NW mice (*p* > 0.05; as determined by unpaired parametric *t*-test).

Statistical analysis was performed using GraphPad Prism v.6.0 software (GraphPad Software, La Jolla, CA, USA). Data in **Figures 1A**, **2A–C**, and **3B–D** were analyzed by two-way ANOVA with Holm-Sidak post-tests. Values in **Figure 2B** were log-transformed prior to statistical analysis. Pre-immune (non-specific) IgG levels of DIO and NW mice were compared using unpaired parametric *t*-test. Comparisons with *p* < 0.05 were considered statistically significant, and are indicated by asterisk (^∗^) in the figures.

## Results

### Antibiotic Treatment of *B. burgdorferi* Infection in DIO Mice

In the present study, we examined whether DIO affected efficacy of treatment with the antibiotic tigecycline in female C3H/HeN mice. Tigecycline was chosen because it has a significantly longer half-life in mice than other antibiotics commonly used for experimental treatment of acute *B. burgdorferi* infection, and is therefore more appropriate for studying treatment efficacy in mice than antibiotics which are commonly used to treat human infection, such as doxycycline and ceftriaxone (Bockenstedt et al., 2002; Hodzic et al., 2008; Barthold et al., 2010). Mice were treated in the acute phase of infection (3 weeks post-inoculation), and antibiotic treatment efficacy was assessed 4 weeks post-treatment (8 weeks post-inoculation). This corresponded to a late, typically post-resolution infection in mock-treated mice (Barthold et al., 1993).

To ensure full infectivity of *B. burgdorferi* used for the experiments, the B31 5A4-derived strain GCB726 (Moriarty et al., 2008, 2012; Javid et al., 2016; Zlotnikov et al., 2016) was passaged through a mouse. The resulting strain was denoted TMB79 (see Materials and Methods for details), and minimum inhibitory and bactericidal concentrations (MIC and MBC) of tigecycline for TMB79 were determined. MIC and MBC values were 0.4 ± 0 and 1.6 ± 0 mg/l, respectively, and were approximately 70- and 2-fold greater than the respective MICs and MBCs for *B. burgdorferi* B31 strains reported in other studies (MIC 0.006–0.016 mg/l and MBC = 0.195–1 mg/l) (Yang et al., 2009; Ates et al., 2010; Barthold et al., 2010). The difference between previously reported MIC values of B31 strains and the MIC value of the TMB79 strain was not caused by the passage through a mouse, since the MICs of tigecycline before and after the passage were identical (0.4 ± 0 mg/l). Tigecycline efficacy in NW mice is best approximated from the pharmacokinetic/pharmacodynamic parameter *f*AUC_0-24_/MIC [area under the free drug concentration-time curve from 0 to 24 h (AUC_0-24_) over the MIC of the pathogen; Crandon et al., 2009]. Based on available studies, *f*AUC_0-24_/MIC in the present study was estimated to be 2.8–5.2 (Crandon et al., 2009; Koomanachai et al., 2009), which corresponds to the tigecycline exposure required to arrest bacterial growth in NW mice (Crandon et al., 2009; Nicasio et al., 2009).

To study the effects of DIO on antibiotic treatment, 80 4-week old female C3H/HeN mice were preconditioned for 12 weeks with either a high fat diet (40 mice) or normal rodent chow (40 mice). After 11 weeks of preconditioning, mice fed a high fat diet weighed 38% more than normal chow controls (NW) (**Figure 1A**), and were therefore obese (DIO). Similar to recent results of previous studies (Shi et al., 2006, 4), DIO was not associated with significant increases in non-fasting blood glucose in female C3H/HeN mice compared to NW control animals after 18 weeks of preconditioning (7.989 ± 0.89 and 6.85 ± 0.66 mmol/l for DIO and NW mice, respectively; *p* > 0.05).

As summarized in the experimental flow chart provided in **Figure 1B**, after 12 weeks of preconditioning, 30 mice in each diet group were infected by needle inoculation at the dorsal lumbar midline with 1 × 10^4^ *B. burgdorferi* strain TMB79. Ten mice in each diet group were mock-infected with *B. burgdorferi* cultivation medium alone (“mock-infected”). Three weeks after *B. burgdorferi* infection (15 weeks from beginning of diet regimens), infected mice in each diet group were further subdivided into 3 groups of 10. Two of these infected groups for each diet were treated with intraperitoneal tigecycline injections every 24 h for 10 days, whereas the third group was injected with tigecycline vehicle alone (10% DMSO in 0.9% saline: “mock-treated”). Similarly, the mock-infected controls were treated with vehicle only. To simulate conditions of antibiotic under-treatment in obesity, NW and DIO infected, antibiotic-treated mice were sub-divided into 2 groups of 10 animals. One each of these NW and DIO groups was treated daily with 406.25 μg of tigecycline (normal weight dose: “NW dose”), which corresponded to a 12.5 mg/kg dose calculated based on the mean weight of NW mice at time of treatment onset (32.5 g). The second of these NW and DIO groups was treated daily with 533.75 μg of tigecycline (“Obese dose”), which corresponded to a 12.5 mg/kg dose calculated based on the mean weight of DIO mice at time of treatment onset (42.7 g).

*Borrelia burgdorferi* is a highly invasive bacterium that can colonize the majority of mouse organs and tissues (Barthold et al., 1990, 1991; Yang et al., 1994; Imai et al., 2013). To assess the effects of DIO and antibiotic treatment on *B. burgdorferi* clearance, hearts and joints were cultivated to recover viable bacteria, and copies of *B. burgdorferi flaB* DNA were quantified in 10 tissues (bladder, brain, ear, heart, kidney, liver, lung, patella/knee joint, quadriceps muscle, and ventral thoracic skin) by qPCR. *B. burgdorferi* infection in mice is accompanied by prominent arthritis and carditis in the joints and heart (Barthold et al., 1990). Hearts were collected at the experimental endpoint for quantitative histological analysis of inflammation (carditis). Severity of arthritis was not measured, since this phenotype typically manifests in juvenile mice infected at 3–5 weeks of age (Barthold et al., 1990), and mice used in the present study were 16-week old adults at time of infection due to the duration of dietary preconditioning. Furthermore, arthritis was not observed in NW and DIO adults infected at a similar age in our recent study (Zlotnikov et al., 2016). Since antibiotic treatment can affect *B. burgdorferi*-specific antibody production (Bockenstedt et al., 2002; Nowakowski et al., 2003; Barthold et al., 2010; Elsner et al., 2015), IgMs and IgGs were measured in sera from all 80 mice collected at four time points: 1 week before infection (“pre-immune”), 2 weeks after infection (“post-inoculation”), 2 weeks after the end of antibiotic treatment (“post-treatment”: 45 days/∼6 weeks post-inoculation), and at the experimental endpoint (4 weeks after the end of antibiotic treatment, 59 days/∼8 weeks post-inoculation).

### Effect of DIO on Tissue Clearance of *B. burgdorferi* DNA in Antibiotic-Treated Mice

Except for one joint of a DIO mock-treated mouse, no viable spirochetes were recovered from hearts and tibiotarsal joints of DIO or NW mice by cultivation. To the best of our knowledge, no previous reports have described cultivation of B31-derived *B. burgdorferi* strains from tissues of immunocompetent mice after 8 weeks of infection with the same or lower infectious doses than the dose used in our study. In addition, most previous studies investigating infection at this time point or later have been performed with the N40 *B. burgdorferi* strain (Bockenstedt et al., 2002, 2012; Yrjänäinen et al., 2007, 2010; Hodzic et al., 2008, 2014). Since individual *B. burgdorferi* strains display differences in infectivity and tissue tropism (Wormser et al., 2008), it is possible that the inability to cultivate viable *B. burgdorferi* from heart and joint at 8 weeks reflected strain-specific differences in colonization of, or viability in, these tissues. Tissues in our study were also cultivated in medium containing gentamicin, since the TMB79 strain is resistant to this antibiotic due to the presence of a selectable plasmid for expression of green fluorescent protein. Although gentamicin resistance was not affected by passage of the TMB79 parent strain GCB726 through a mouse after 3 weeks of infection, it is possible that the loss of this plasmid during infection prevented recovery of live bacteria at 8 weeks post-infection.

Due to the extremely low rate of recovery of viable bacteria from hearts and joints at this infection stage, we therefore monitored clearance of bacterial DNA from tissues by measuring *B. burgdorferi flaB* DNA copy number (**Figure 2**, **Table 1**). To ensure that copy number measurements did not include false-positive values arising from stochastic amplification of potentially contaminating *B. burgdorferi flaB* DNA sequences or of other *flaB*-like sequences with similar melt-curve profiles, qPCR was performed in tissue samples from mock-infected mice, and the background values (mean plus 2 standard deviations) were subtracted for each tissue from the values for samples for the same tissue from infected animals. The resulting *flaB* copy number values were normalized to total μg of DNA in each reaction, to control for differences in cell density and DNA extraction efficiency among tissues and individual samples.

**Table 1:** Number of tissues positive for the presence of *B. burgdorferi flaB* DNA, as determined by qPCR.

	DIO			NW		
Treatment group	Mock-treated	“NW dose”	“Obese dose”	Mock-treated	“NW dose”	“Obese dose”
Heart	5/10	0/10	0/10	1/10	0/10	0/10
Ear	10/10	3/10	3/10	10/10	1/10	4/10
Skin	9/10	0/10	0/10	10/10	0/10	0/10
Lung	3/10	0/10	0/10	2/10	0/10	0/10
Bladder	9/10	1/10	0/10	9/10	0/10	0/10
Brain	5/9	4/10	4/10	3/10	8/10	4/10
Kidney	1/10	5/10	0/10	1/10	5/10	0/10
Knee joint	8/10	3/8	2/10	10/10	0/10	5/10
Liver	0/10	1/10	0/10	0/10	1/10	2/10
Quadriceps	0/10	0/10	1/10	1/10	0/10	0/10

*Results are summarized as number of positive tissues per number of analyzed individual tissues in each experimental group. Raw data for all tissues are provided in Supplementary Figures S1 and S2. All tissues of DIO and NW mock-infected mice were qPCR negative*.

The percentage of *flaB*-positive tissues/mouse (**Figure 2A**) and median *flaB* DNA copy number per tissue (**Figure 2B**) were comparable in DIO and NW mock-treated mice. At 4-weeks post-inoculation, the *flaB* DNA copy number is most commonly elevated in heart, brain, liver, lung, knee joint of DIO mice (Zlotnikov et al., 2016). In the present study, the only tissue with a greater *flaB* DNA copy number in mock-treated DIO animals was heart (median 1,825-fold and mean 5.4-fold; Supplementary Figure S1A). In contrast, the bacterial burden was elevated in the ears of NW mock-treated mice when compared to their DIO counterparts (median 3.9-fold and mean 3.5-fold; Supplementary Figure S1B). Therefore, by a late stage of infection (8 weeks post-inoculation), DIO in female mice was associated with elevated bacterial DNA burden in heart only.

Despite the large fold difference in *flaB* DNA copy number in the hearts of mock-treated DIO and NW mice, no carditis was observed in any experimental group, as measured using a sensitive nuclei counting-based carditis quantification method (**Figures 2C,D**; Supplementary Figures S3 and S4) (Javid et al., 2016). Carditis in *B. burgdorferi*-infected mice typically peaks at 2–4 weeks post-inoculation and declines thereafter (Armstrong et al., 1992; Barthold et al., 1993; Bockenstedt et al., 2001; Lasky et al., 2015), and it is likely that carditis had already resolved by the endpoint of our studies. In mice fed a high fat diet for prolonged periods, cardiomyocyte hypertrophy and accumulation of extracellular matrix can also lead to reduced cellular density in the heart (Wang et al., 2015). However, in the present study, significant hypocellularity in the hearts of DIO mice was not observed (**Figures 2C,D**; Supplementary Figures S3 and S4).

Across doses, tigecycline treatment resulted in a 73% and a 68% reduction in the percentage of *flaB*-positive tissues per mouse compared to DIO and NW mock-treated animals, respectively (*p* < 0.05 for treated compared to mock-treated) (**Figure 2A**). Full clearance from all tissues, determined by identifying mice with no *flaB*-positive tissues, was observed in 1/20 (5%) DIO and 4/20 (20%) NW antibiotic-treated mice, and did not differ significantly between diet groups (*p* > 0.05). Across doses, the average *flaB* DNA copy number/tissue was reduced by 256-fold and 95–fold respectively in tigecycline-treated DIO and NW animals compared to mock-treated controls (*p* < 0.05) (**Figure 2B**). The percentage of *flaB*-positive tissues/mouse (**Figure 2A**) and median *flaB* copy number per tissue (**Figure 2B**) also did not significantly differ among any experimental groups following treatment with either “NW” or “Obese” antibiotic doses. These results indicated that in the context of whole mice (all 10 examined tissues) DIO did not affect tigecycline-dependent clearance of *B. burgdorferi* DNA, and that NW and obese weight doses were similarly effective. Since no viable bacteria were recovered from any tissues, the DNA measured in tissues could have been derived from uncleared bacterial debris (Bockenstedt et al., 2012), or uncultivatable bacteria remaining in tissues (Hodzic et al., 2014). However, since DIO did not affect antibiotic-dependent *B. burgdorferi* DNA clearance, additional experiments using previously described transcription- or xenodiagnosis-based methods (Bockenstedt et al., 2002; Hodzic et al., 2008) were not performed to determine if the DNA remaining in tissues was derived from live bacteria.

At the level of individual tissues, heart (Supplementary Figure S1A), skin (Supplementary Figure S1C) and lung (Supplementary Figure S1D) were the only tissues completely cleared of *B. burgdorferi* DNA after tigecycline treatment in all experimental groups. In bladder, all but one of the DIO mice were qPCR negative after antibiotic treatment (Supplementary Figure S1E). In brain (Supplementary Figure S1F), DNA copy number was comparable to or greater than mock-treated mice in most antibiotic-treated groups, suggesting that tigecycline treatment was not effective in this organ. In kidney (Supplementary Figure S2A), copy number was higher in both NW and DIO mice treated with the NW dose of tigecycline than in mock-treated controls, whereas *B. burgdorferi* DNA was completely cleared in kidneys following treatment with the higher, “Obese dose” of tigecycline. In the knee joint (Supplementary Figure S2B) and ear (Supplementary Figure S1B), antibiotic treatment significantly reduced but failed to clear *B. burgdorferi* DNA in most groups. Liver (Supplementary Figure S2C) and quadriceps muscle (Supplementary Figure S2D) were negative for *B. burgdorferi* DNA in 9/10 mock-treated mice from both diet groups by 8 weeks post-infection, and copy number did not differ significantly in mock-treated and treated groups. Unlike in kidney, where 9/10 untreated mice were *flaB*-negative (Supplementary Figure S2A), tigecycline treatment did not result in significantly increased copy number in liver or quadriceps muscle (Supplementary Figures S2C,D). Collectively, these results implied that DIO did not reduce tigecycline treatment efficacy, but that, similar to previous reports (Bockenstedt et al., 2002, 2012; Hodzic et al., 2008, 2014; Barthold et al., 2010; Yrjänäinen et al., 2010), antibiotic treatment itself did not eliminate *B. burgdorferi* DNA from several tissues, including brain, kidney, knee joint and ear.

### Effects of DIO on *B. burgdorferi*-Specific Antibody Production in Untreated and Antibiotic-Treated Mice

Finally, effects of DIO on *B. burgdorferi*-specific IgM and IgG production were measured over the course of infection by ELISA, using whole cell lysates of the same *B. burgdorferi* strain used for infection as the ELISA antigen (**Figure 3**; Supplementary Figure S5). Sera of mock-infected mice did not contain *B. burgdorferi*-reactive IgMs or IgGs at any time point. As expected (Barthold et al., 1991), *B. burgdorferi*-specific IgM levels progressively declined through the course of the experiment in both mock-treated and treated groups (Supplementary Figure S5). The kinetics of IgG production in various experimental groups were compared by normalizing ELISA absorbance values to the median absorbance value for infected, mock-treated NW mice at the experimental endpoint (8 weeks after infection), after correcting for differences in ELISA serum dilutions at different time points (**Figure 3A**; raw data and results of statistical analysis are shown in **Figures 3B–D**). *B. burgdorferi*-specific IgG production in mock-treated DIO mice was delayed and attenuated compared to NW counterparts within the 8-week study period (**Figure 3A**). *B. burgdorferi*-specific IgG levels were lower in mock-treated infected DIO mice than in NW counterparts at 6 and 8 weeks post-inoculation (**Figures 3C,D**), indicating that production of antibodies in response to *B. burgdorferi* was impaired in DIO.

In mice and humans, antibiotic treatment in Lyme disease can result in reduced circulating IgG levels (Bockenstedt et al., 2002; Nowakowski et al., 2003; Hodzic et al., 2008, 2014; Barthold et al., 2010; Elsner et al., 2015). In mice, this is due to *B. burgdorferi*-suppressed formation of the long-lived germinal centers required for maintaining long-lived humoral immunity, and dependence of antibody production on continued antigen stimulation-based development of short-lived germinal centers (Elsner et al., 2015). Similarly, *B. burgdorferi*-specific IgGs in both NW and DIO mice were lower following antibiotic treatment than in mock-treated controls at both 2 and 4 weeks post-treatment (6 and 8 weeks post-infection) (**Figures 3C,D**). Therefore, tigecycline treatment led to attenuated *B. burgdorferi*-specific IgG production, likely due to reduction of the amount of bacterial antigen driving humoral immune responses. This is supported by a strong correlation between IgG levels at both 6 and 8 weeks post-inoculation (2 and 4 weeks post-treatment) and a total number of *flaB* DNA copies in tissues of *B. burgdorferi*-infected mice across diet and treatment groups (both *p <* 0.0001, as determined by Spearman correlation). Serum IgG levels were similar in all groups of tigecycline-treated mice (*p* > 0.05; **Figures 3C,D**), indicating that neither DIO nor antibiotic dose significantly affected IgG production in treated animals. Collectively, these results indicated that production of *B. burgdorferi*-specific IgGs was delayed and attenuated in DIO and that antibiotic treatment inhibited generation of specific IgGs in both diet groups, but that the inhibitory effects of DIO and tigecycline treatment on IgG production were not additive.

## Discussion

In this study, we found that DIO does not affect tigecycline-dependent clearance of *B. burgdorferi* DNA from tissues of female C3H/HeN mice, and that calibrating treatment dose to average body mass does not significantly alter clearance efficacy. Thus, DIO does not impair the efficacy of tigecycline treatment initiated at an acute phase of *B. burgdorferi* infection. These results provide valuable information about the efficacy of tigecycline treatment in facilitating clearance of bacterial DNA from a range of tissues in both NW and DIO mice, as well as further insight into the effects of DIO on immune responses to *B. burgdorferi* infection.

In the acute stage of *B. burgdorferi* infection in female mice (4 weeks post-inoculation), tissues affected by DIO include heart, brain, knee joint, liver and lung (Zlotnikov et al., 2016). By 8 weeks *B. burgdorferi* DNA had been largely cleared from liver and lung in both NW and DIO groups, and there was no significant difference in bacterial burden in the knee joint and brain between DIO and NW mice. Therefore, the heart was the only organ where impaired *B. burgdorferi* clearance in DIO continued into late infection stages in female animals. Although we and others have found that metabolic disorders such as obesity-independent hyperglycemia and hypercholesterolemia in mice inhibit control of *B. burgdorferi* burden and/or tissue clearance of bacterial DNA (Toledo et al., 2015; Javid et al., 2016), female DIO mice were not hyperglycemic (Shi et al., 2006), and DIO alone does not induce hypercholesterolemia in mice without concomitant deficiencies in apolipoprotein E or low density lipoprotein receptor (Karagiannides et al., 2008). Thus, increased burden in the heart at late infection stages was not due to hyperglycemia or hypercholesterolemia. Passive transfer of immune serum to *B. burgdorferi* facilitates clearance of some but not all bacterial DNA from heart (Barthold et al., 2006), suggesting that deficits in IgG production in female mice with DIO likely contributed to elevated burden in this tissue. A similar effect of DIO on specific IgG production and control of bacterial load has recently been reported for *S. aureus* infections in mice (Farnsworth et al., 2015).

Our data showed that tigecycline treatment did not significantly reduce *B. burgdorferi* DNA copy number in brain. Comparable to other antibiotics, most of which do not cross the blood-brain barrier efficiently, tigecycline concentration in the brain is approximately 10 times lower than in serum (Nau et al., 2010). It is therefore possible that tigecycline did not reach sufficiently high concentrations in the brain to reduce bacterial load in this organ. Furthermore, treatment with the “NW dose” but not the “Obese dose” of tigecycline resulted in significantly increased bacterial DNA burden in kidney compared to mock-treated mice. Higher doses of this antibiotic may have been required for effective antimicrobial treatment in kidneys. Nonetheless, this hypothesis does not explain why bacterial DNA burden was greater in kidneys of mice treated with a normal-weight dose of tigecycline than in untreated mice, where kidneys were largely negative for *B. burgdorferi* DNA. Tigecycline has not been associated with significant clinical nephrotoxicity (Estes and Derendorf, 2010). However, one possibility is that lower dose tigecycline treatment affected kidney microstructure or function, rendering this organ more sensitive to bacterial colonization, or less susceptible to clearance of bacteria and their debris by phagocytic cells, but that this colonization/persistence advantage was eradicated at a higher drug dose. Since we did not examine pathology in kidney, which does not develop prominent pathology in inbred laboratory mouse strains infected with *B. burgdorferi* (Schaible et al., 1989; Barthold et al., 1990, 1991; Schneider et al., 2013), we do not know if the presence of bacterial DNA in these organs was clinically meaningful, or if tigecycline treatment was associated with histological changes which might affect bacterial colonization and persistence.

Persistence of *B. burgdorferi* in tissues has been explored in numerous studies using various antibiotic combinations, different animal models and distinct *B. burgdorferi* strains (Straubinger et al., 1997; Hodzic et al., 2008, 2014; Barthold et al., 2010; Yrjänäinen et al., 2010; Bockenstedt et al., 2012; Embers et al., 2012; Pavia and Wormser, 2014). In the majority of cases, bacterial DNA can be detected in animal tissues post-antibiotic treatment; however, controversy exists on viability and infectious status of persistent bacteria (Wormser and Schwartz, 2009; Bockenstedt and Radolf, 2014; Pavia and Wormser, 2014). Bacteria were not recovered by culture in experiments described here, and we cannot therefore comment on the effect of antibiotics on persistence and infectivity of live *B. burgdorferi* in the context of DIO. Bacterial DNA was detected in 14% (27/198) and 15% (30/199) of tissues from tigecycline-treated DIO and NW mice, respectively, which is comparable to the percentage of qPCR-positive tissues in immunocompetent mice treated by ceftriaxone or tigecycline reported in previous studies (Hodzic et al., 2008; Barthold et al., 2010). Furthermore, Barthold et al. (2010) observed 27% (11/40) qPCR positive-tissues in mice treated with a four-times higher (50 mg/kg) dose of tigecycline than was used in the present study, and it is therefore unlikely that increasing the tigecycline dose would change outcomes of treatment in DIO or NW mice. In contrast, lowering the tigecycline dose might alter treatment outcomes in both DIO and NW mice. However, potential differences between bacterial burden in DIO and NW mice would likely stem from ineffective dosing rather than the effects of DIO; as estimated from the value of fAUC_0-24_/MIC, a lower tigecycline dose would likely not be effective against the *B. burgdorferi* strain TMB79 used in this study (Crandon et al., 2009; Koomanachai et al., 2009). Alternative results might have been also observed if DIO mice were treated with another antibiotic, since obesity can affect pharmacokinetic and pharmacodynamic parameters of individual drugs differently (Falagas and Karageorgopoulos, 2010).

In the present study, DIO mice displayed delayed and attenuated production of *B. burgdorferi*-specific IgGs within the 8-week period of this study. Obesity is known to reduce the efficacy of vaccination against a number of viral pathogens, and to impair antibody production in response to influenza vaccination, T cell-independent antigen and *S. aureus* infection (Sheridan et al., 2012; Teague et al., 2013; Farnsworth et al., 2015; Painter et al., 2015). Longer infectivity studies in DIO mice are warranted to explore the possibility that impaired *B. burgdorferi*-specific IgG production in DIO might contribute to increased susceptibility to *B. burgdorferi* reinfection (Nadelman et al., 2012; Rogovskyy and Bankhead, 2013; Elsner et al., 2015).

## Conclusion

Here we showed that DIO does not impair tigecycline efficacy in treating *B. burgdorferi* infection. Furthermore, our data indicate that DIO suppresses humoral immune responses to this pathogen, warranting further investigation of the mechanisms and possible consequences of this immune suppression in animal models of DIO and human populations.

## Author Contributions

Conceived and designed the experiments: HP and TM; Performed the experiments: HP, AE, and ZA; Analyzed the data: HP, NZ, and TM; Contributed reagents/materials/analysis tools: TM and CC; Prepared the original draft: HP, AE, and TM; Reviewed and edited the manuscript: NZ, ZA, and CC.

## Conflict of Interest Statement

The authors declare that the research was conducted in the absence of any commercial or financial relationships that could be construed as a potential conflict of interest.
