# Supplementary material for: Diet-Induced Obesity Does Not Alter Tigecycline Treatment Efficacy in Murine Lyme Disease
Source: Front Microbiol. 2017 Feb 24;8:292. doi: 10.3389/fmicb.2017.00292 (PMC5323460; doi:10.3389/fmicb.2017.00292)
Supplement: Supplementary file 1 [file Image_1.PDF]

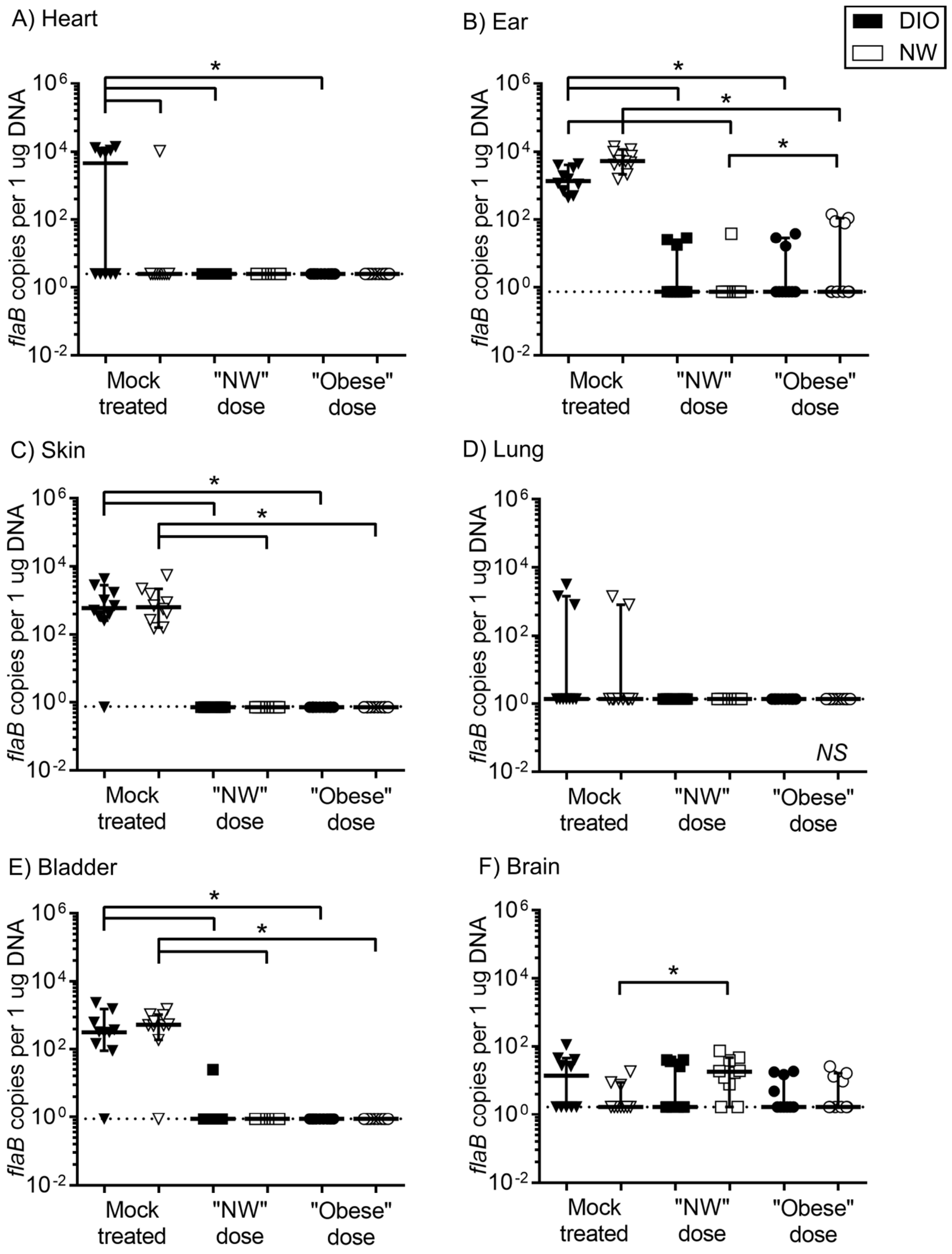

Supplementary Figure 1. *B. burgdorferi flaB* DNA copy number in individual tissues. Bacterial DNA in indicated tissues was quantified by qPCR with primers specific to the *B. burgdorferi flaB* DNA sequence 4 weeks post-treatment with antibiotics (8 weeks post-inoculation). *flaB* DNA copy number in each sample was normalized to 1 µg of total host and bacterial DNA in the sample. Bars represent medians with 95% CI. * indicates $p<0.05$ (two-way ANOVA of log-transformed values with Holm-Sidak post-tests). **A.** Heart. **B.** Ear. **C.** Skin. **D.** Lung. **E.** Bladder. **F.** Brain.
