# Supplementary material for: Diet-Induced Obesity Does Not Alter Tigecycline Treatment Efficacy in Murine Lyme Disease
Source: Front Microbiol. 2017 Feb 24;8:292. doi: 10.3389/fmicb.2017.00292 (PMC5323460; doi:10.3389/fmicb.2017.00292)
Supplement: Supplementary file 2 [file Image_2.PDF]

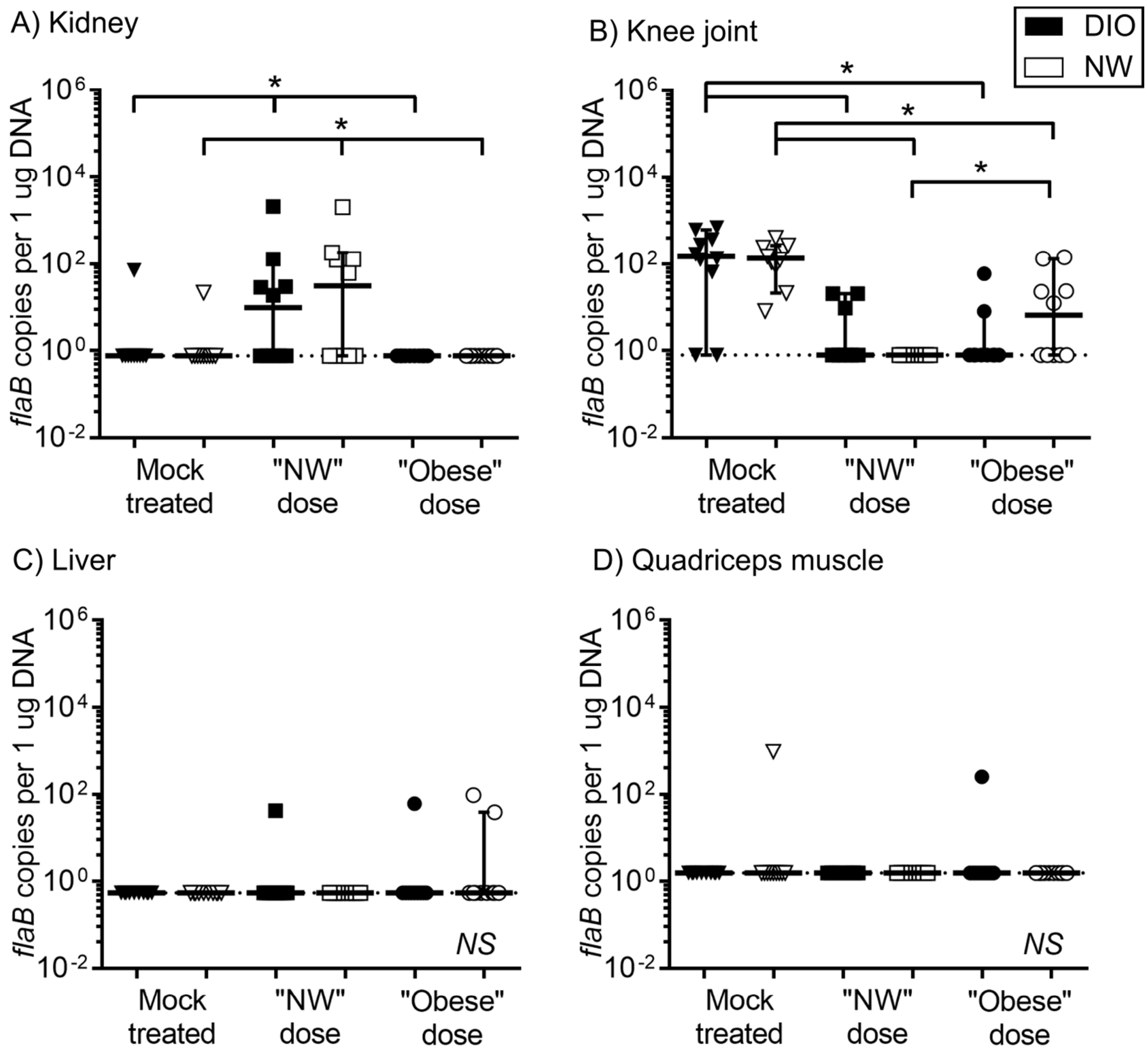

Figure S2. ***B. burgdorferi flaB*** **DNA copy number in individual tissues (continued).** Copy number values for additional tissues not presented in **Fig. S1**. **A.** Kidney **B.** Knee joint. **C.** Liver. **D.** Quadriceps muscle.
