# Supplementary material for: Diet-Induced Obesity Does Not Alter Tigecycline Treatment Efficacy in Murine Lyme Disease
Source: Front Microbiol. 2017 Feb 24;8:292. doi: 10.3389/fmicb.2017.00292 (PMC5323460; doi:10.3389/fmicb.2017.00292)
Supplement: Supplementary file 3 [file Image_3.PDF]

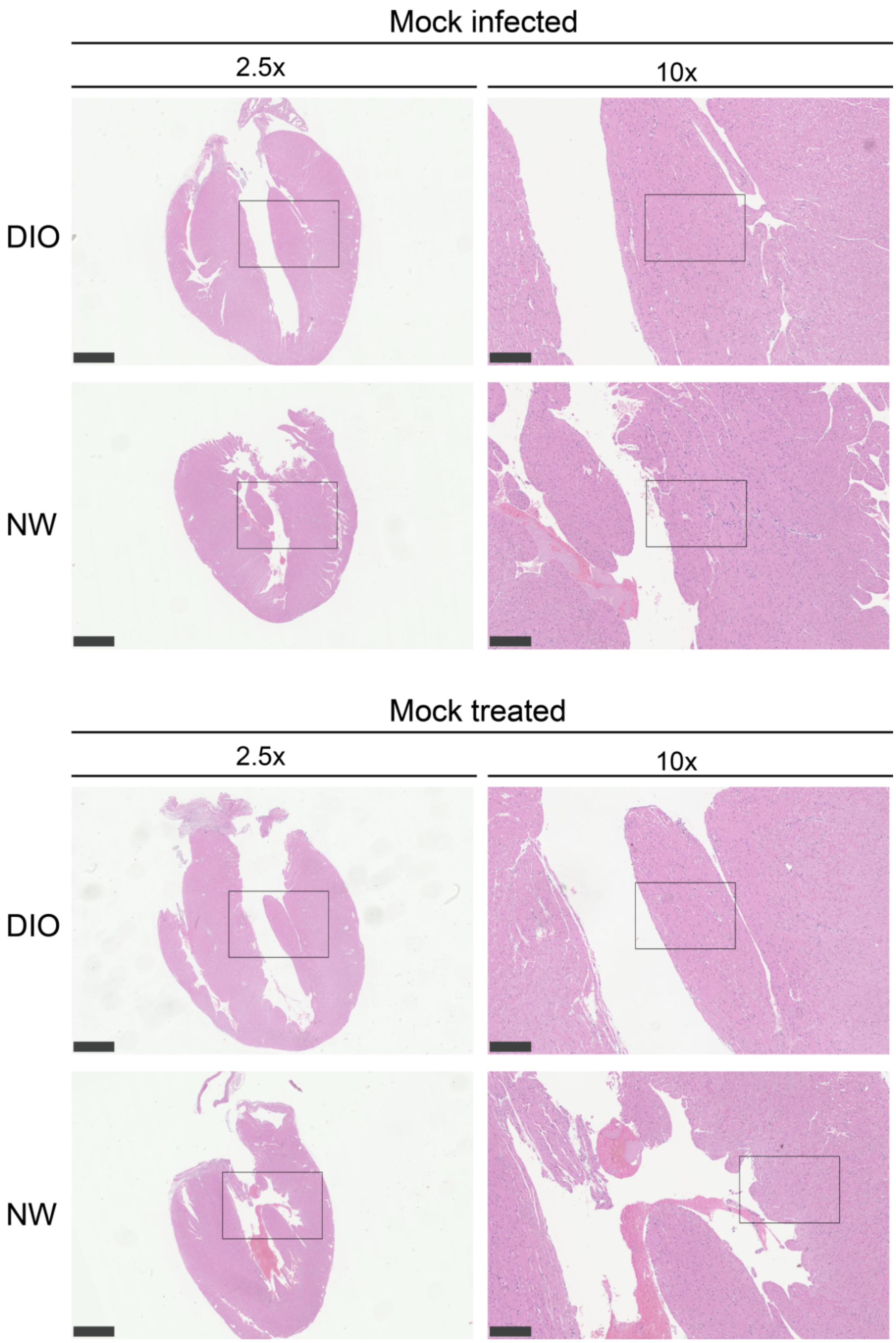

Figure S3. Representative heart sections from mock-infected and mock-treated mice. Black rectangles outline regions depicted in the following magnifications. Bars correspond to 1 mm (2.5x magnification; on the left) and 250 µm (10x magnification; on the right).
