# Supplementary material for: Diet-Induced Obesity Does Not Alter Tigecycline Treatment Efficacy in Murine Lyme Disease
Source: Front Microbiol. 2017 Feb 24;8:292. doi: 10.3389/fmicb.2017.00292 (PMC5323460; doi:10.3389/fmicb.2017.00292)
Supplement: Supplementary file 5 [file Image_5.PDF]

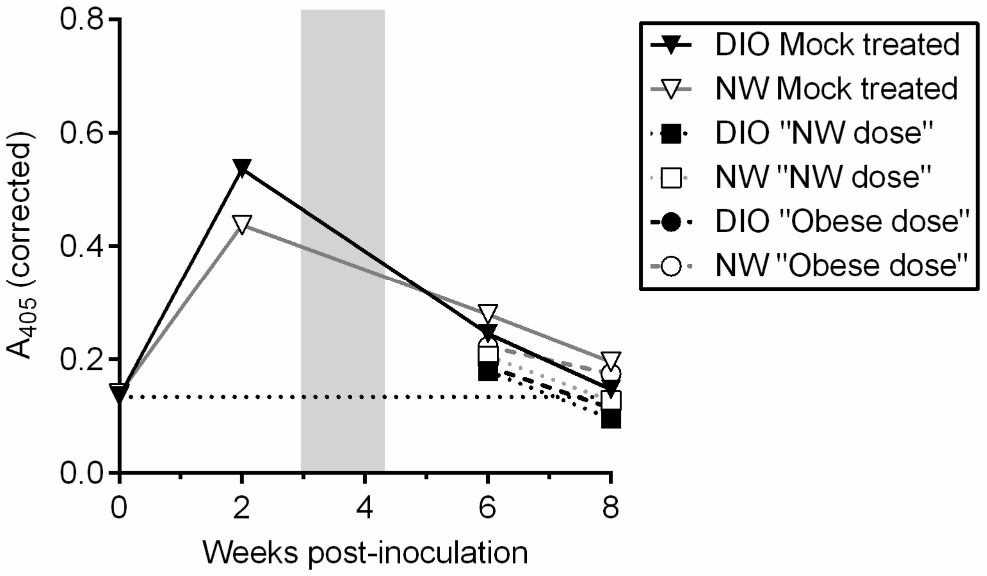

Figure S5. ***B. burgdorferi*-specific serum IgMs.** Development of *B. burgdorferi*-specific IgM responses in DIO and NW mice during infection and tigecycline treatment. Median values for DIO and NW mice are shown in black (with black lines) and white (with grey lines), respectively. The shaded area represents the antibiotic treatment period. Due to the small serum volumes collected at 2 and 6 weeks post-inoculation, samples for IgM analysis were physically pooled before ELISA. This procedure may have masked variation to highly reactive or non-reactive samples. IgM levels did not differ significantly in DIO and NW mice at any time point ($p>0.05$; two-way ANOVA with Holm-Sidak post-tests). Pre-immune sera from individual mice were used as a negative control at 1:100 dilution (dotted line). Levels of pre-immune (non-specific) IgM levels did not differ between DIO and NW mice ($p>0.05$; as determined by unpaired parametric t-test).
